# Supplementary material for: Ligand recognition and activation of neuromedin U receptor 2
Source: Nat Commun. 2022 Dec 27;13:7955. doi: 10.1038/s41467-022-34814-4 (PMC9794833; doi:10.1038/s41467-022-34814-4)
Supplement: Supplementary file 1 — Supplementary Information [file 41467_2022_34814_MOESM1_ESM.pdf]

Supplementary Information

for

Ligand recognition and activation of neuromedin U receptor 2

Wenli Zhao, Wenru Zhang, Mu Wang, Minmin Lu, et al.

Supplementary Figures

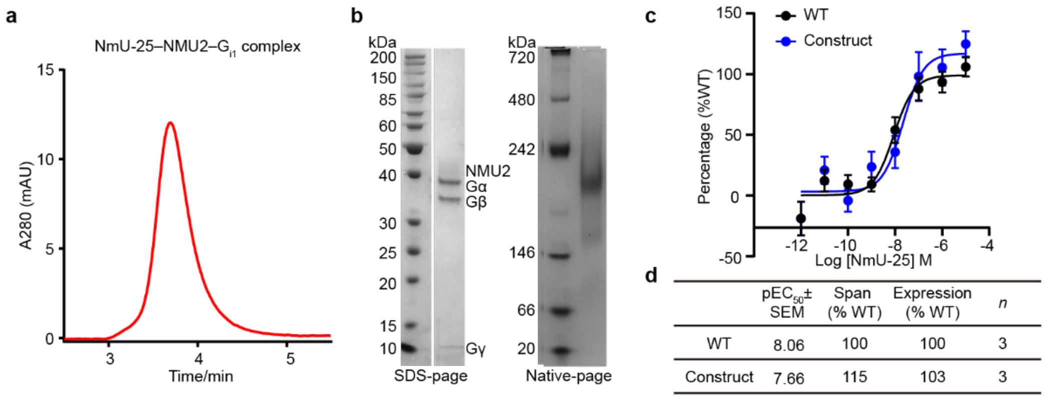

	pEC50± SEM	Span (% WT)	Expression (% WT)	*n*
WT	8.06	100	100	3
Construct	7.66	115	103	3

Supplementary Fig. 1 Complex purification and functional validation of the NmU-25–NMU2–G_{i1} complex. a Analytical size-exclusion chromatography of the purified NmU-25–NMU2–G_{i1} complex. **b** SDS-page and native-page of the purified complex. Gel source data are provided as a Supplementary Information file. **c** Functional validation of structural construct for NmU-25–induced G protein activation by TRUPATH sensor. Data are shown as mean from three independent experiments performed. **d** The span is defined as the window between the maximal agonist response (E_{max}) and minimal agonist response. N represents the number of independent experiments. Source data are provided as a Source Data file.

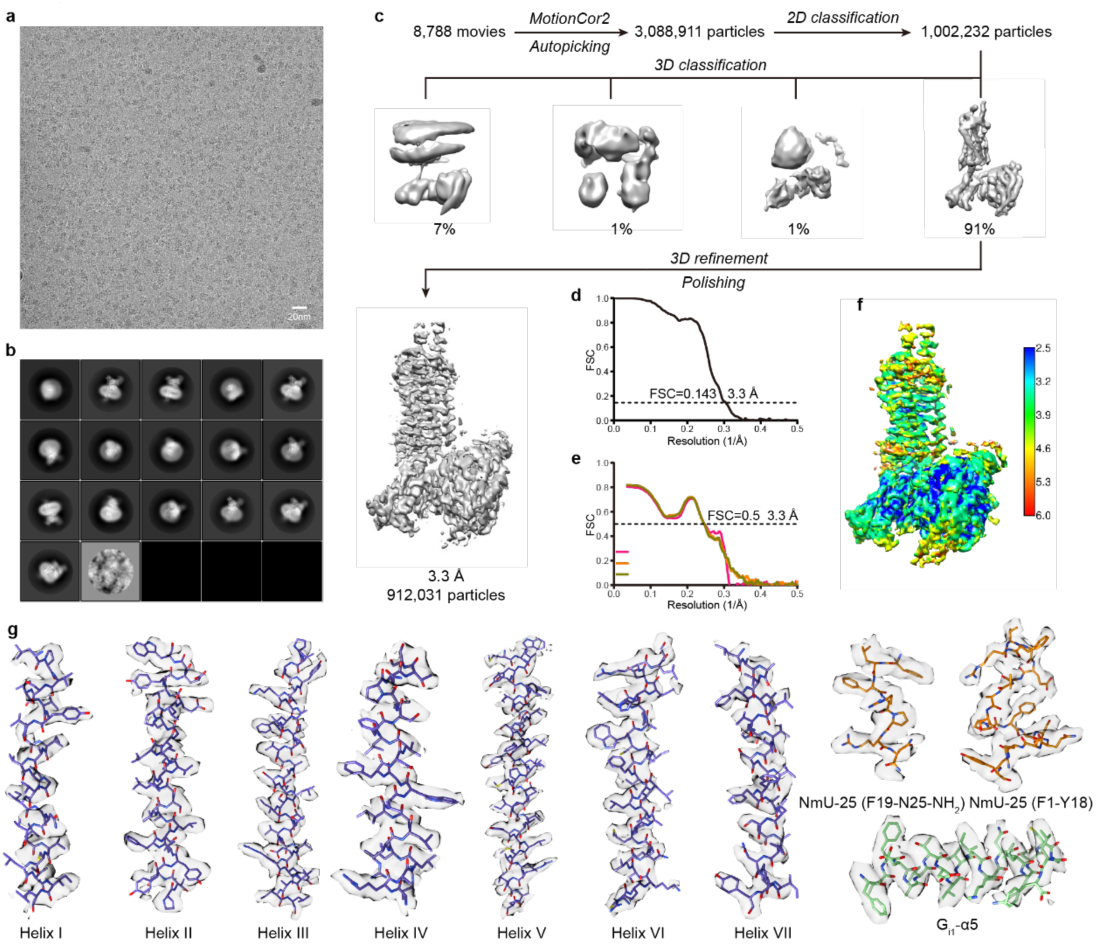

Supplementary Fig. 2 Sample preparation and cryo-EM data procession. a Representative cryo-EM image during the data collection. **b** 2D class average image of the NmU-25–NMU2–G_{i1} complex. **c** Cryo-EM data procession flow chart of the NmU-25–NMU2–G_{i1} complex. **d** Gold-standard FSC curve showing an overall resolution is 3.3 Å at FSC=0.143. **e** Cross-validation of model to density map. **f** Density map according to local resolution estimation. **g** Cryo-EM density map and fitted model of all transmembrane helices of NMU2, NmU-25 and α5 helix of $G\alpha_{i1}$. NMU2, NmU-25 and α5 helix of $G\alpha_{i1}$ are shown as sticks representation and colored by slate, orange and light green.

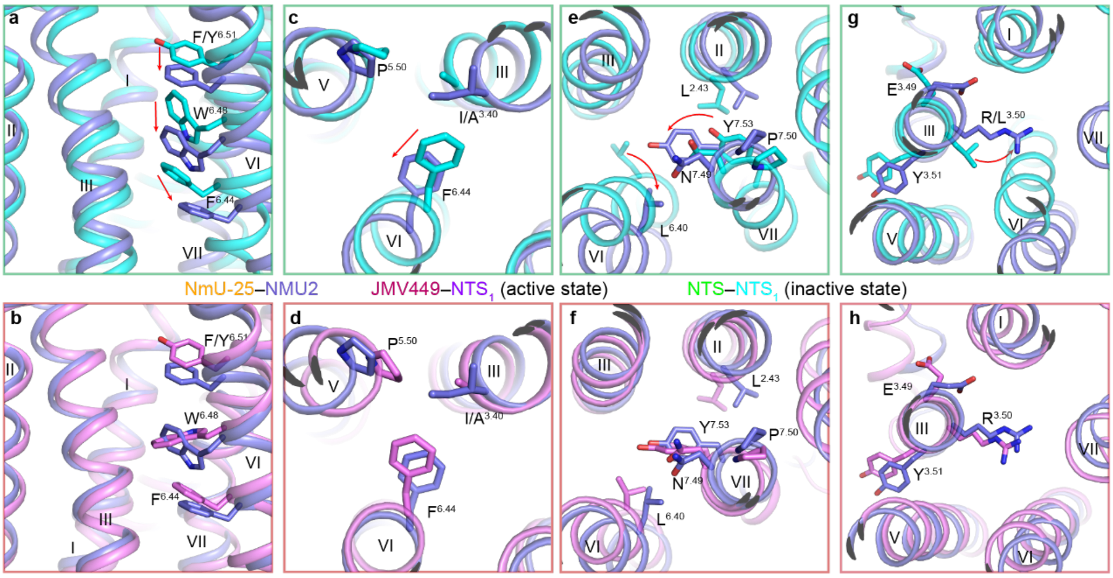

Supplementary Fig. 3 Active conformation of NMU2. a-h Structural comparison between NMU2, active NTS_1 (PDB ID: 6OS9) and inactive NTS_1 (PDB ID: 4BUO). NMU2, active NTS_1 and inactive NTS_1 are shown as cartoon and colored by slate, violet and cyan respectively. Compared with inactive and active NTS_1, the dislocation of the "toggle switch" (**a**, **b**), "PIF" motif (**c**, **d**), "NPxxY" motif (**e**, **f**) and "ERY" motif (**g**, **h**) during the NMU2 activation are indicated by red arrows. Residues are shown as sticks and colored by slate (NMU2), cyan (inactive NTS_1) and violet (active NTS_1).

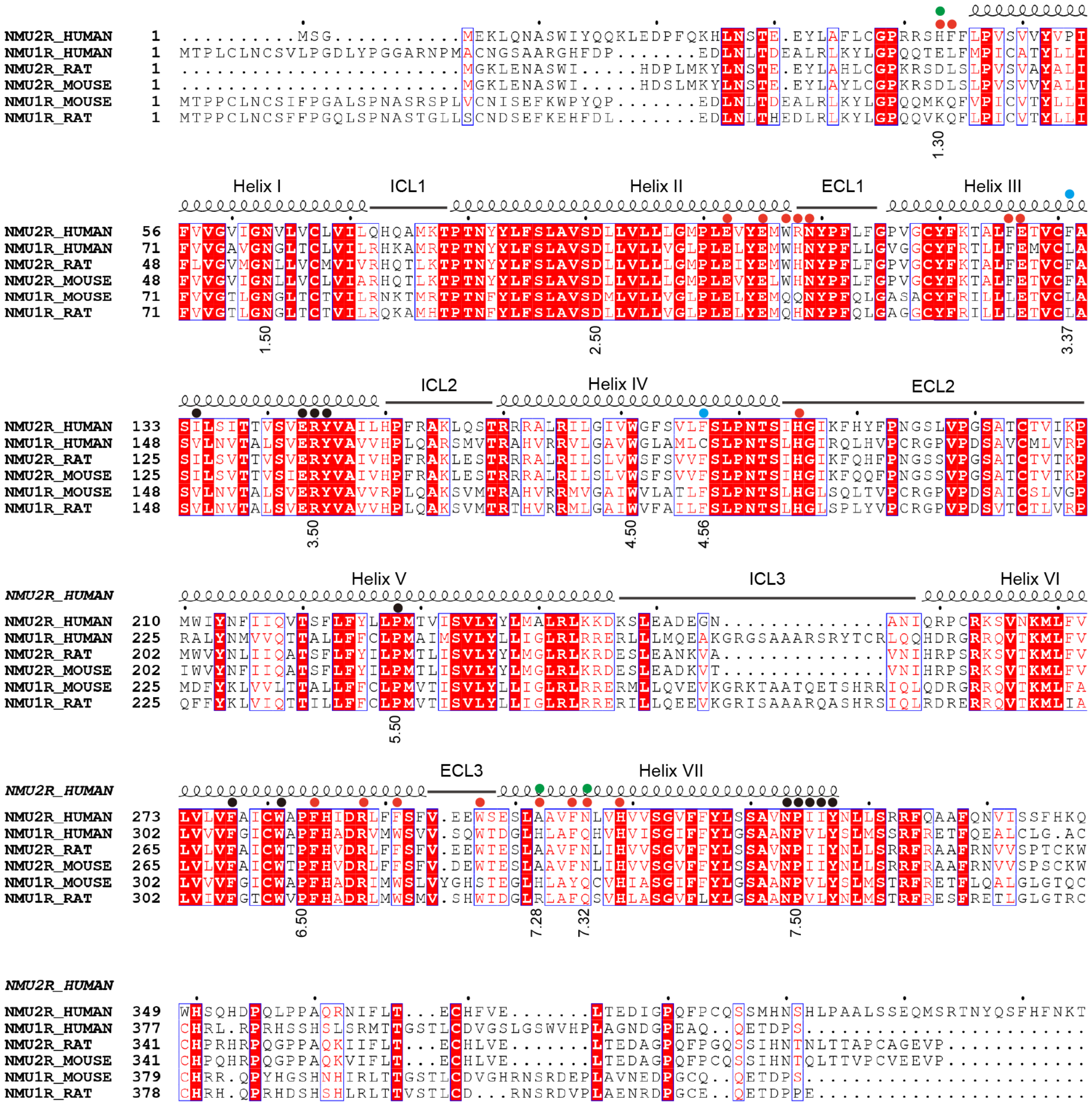

Supplementary Fig. 4 Sequence alignment of NMUs. Sequence alignment was prepared by uniport (https://www.uniprot.org/) and the graphic was generated by ESPript 3.0 server (https://espript.ibcp.fr/ESPript/). Background colors represent the degree of similarity between different receptors: red, identical; red text, similar. Key residues involved in ligand binding are indicated by different color dots above the sequence alignment respectively. Red dots indicate residues involved in NmU-25 binding; green dots indicate specific residues involved in CPN 116 binding; blue dots indicate residues involved in R-PSOP binding; black dots indicate residues involved in the receptor activation.

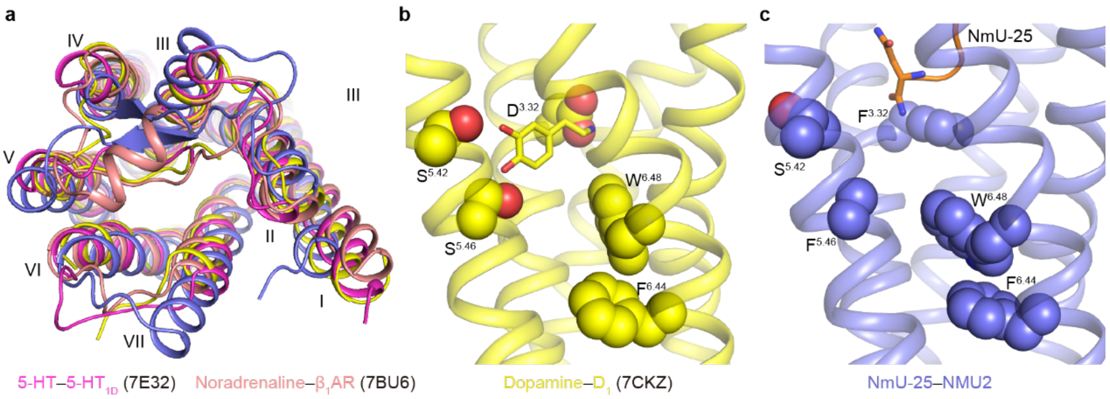

Supplementary Fig. 5 Structural superposition of helical core of NMU2 with other active state GPCRs. a Structural superimpose of monoamine receptors (5-HT–5-HT_{1D}, 7E32; dopamine–D_1, 7CKZ; noradrenaline–β_1AR, 7BU6) with NMU2 from extracellular view. Receptors are shown as light magenta (5-HT_{1D}), yellow (D_1), salmon (β_1AR) and slate (NMU2) cartoon. **b-c** Conformations of the $D^{3.32}$-$S^{5.42}$-$S^{5.46}$ motif, $W^{6.48}$ and $F^{6.44}$ of monoamine receptor (**b**) and NMU2 (**c**). Side chains of residues are shown as sphere. Dopamine is shown as yellow sticks, NmU-25 is shown as orange sticks and cartoon.

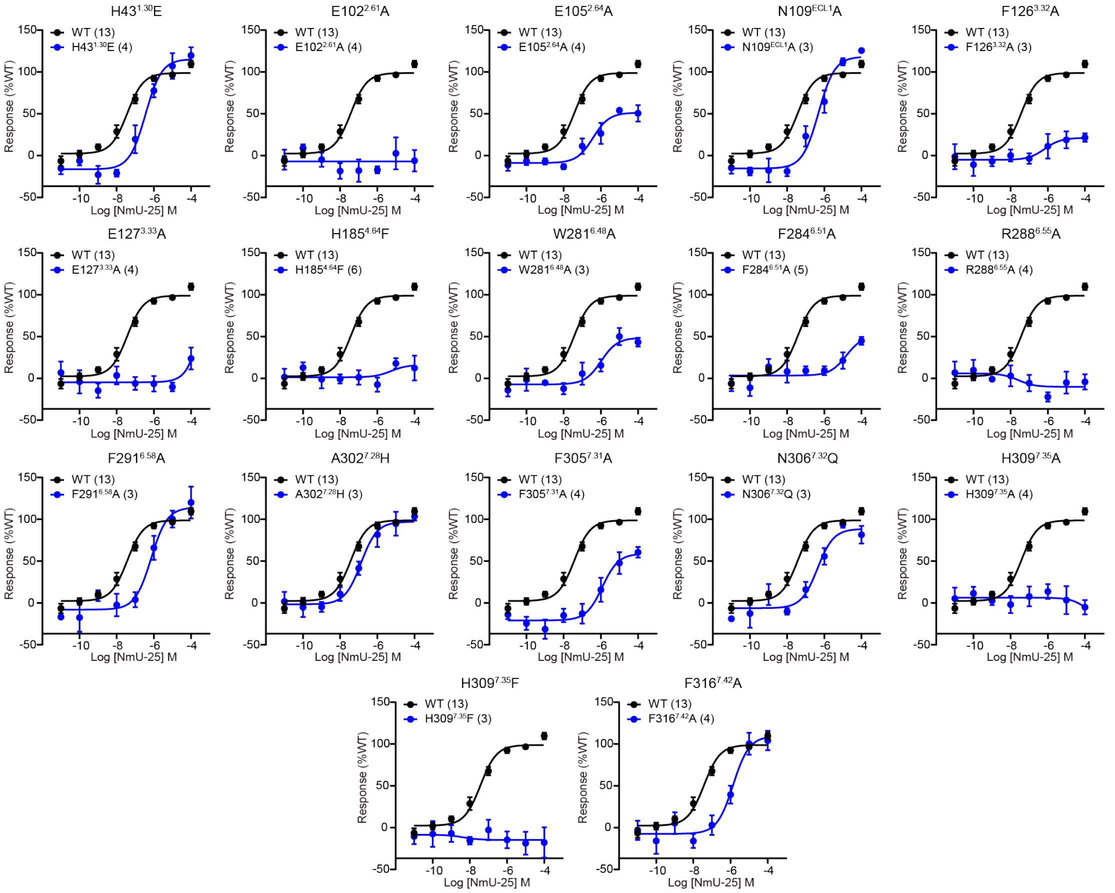

Supplementary Fig. 6 NmU-25–induced G protein activation of NMU2. Dose-response curves (10^{-11} – 10^{-4} M) of NmU-25 on NMU2 reflect the effects of the mutants on receptor's signaling. Data are shown as mean ± SEM from at least three independent experiments. The numbers of independent experiments are shown in the parentheses. Supplementary Table 2 provides detailed statistical evaluation, *P* values, numbers of independent experiments (*n*) and expression levels. Source data are provided as a Source Data file.

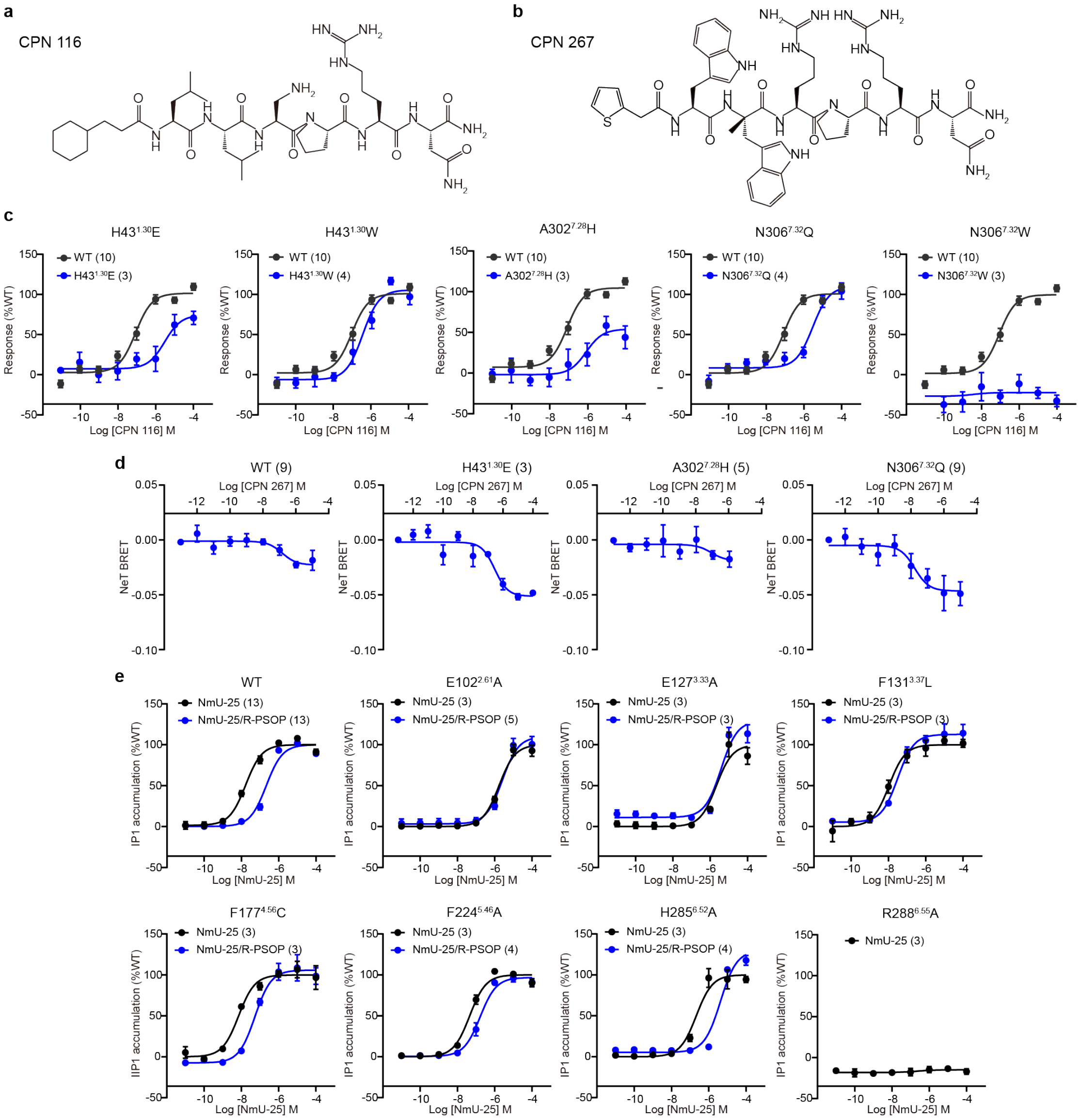

Supplementary Fig. 7 Functional validation of selective ligands. a Chemical structure of selective NmU analogs (CPN 116) of NMU2[1]. **b** Chemical structure of selective NmU analogs (CPN 267) of NMU1[2]. **c** Dose-response curves (10^{-11} – 10^{-4} M) of CPN 116 on NMU2 reflect the effects of the mutants on receptor's signaling. Data are shown as mean ± SEM from at least three independent experiments. The numbers of independent experiments are shown in the parentheses. Supplementary Table 2 provides detailed statistical evaluation, *P* values, numbers of independent experiments (*n*) and expression levels. Source data are provided as a Source Data file. **d** Dose-response curves (10^{-13} – 10^{-4} M) of CPN 267 on NMU2 reflect the effects of the mutants on receptor's signaling. Data are shown as mean ± SEM from at least three independent experiments. Supplementary Table 2 provides detailed statistical evaluation, *P* values,

numbers of independent experiments (*n*) and expression levels. Source data are provided as a Source Data file. **e** IP accumulation of WT and mutants induced by NmU-25 (10^{-11} – 10^{-4} M, black) or NmU-25 (10^{-11} – 10^{-4} M) with R-PSOP (10 μM, blue). Data are shown as mean ± SEM from at least three independent experiments. Supplementary Table 3 provides detailed statistical evaluation, *P* values, numbers of independent experiments (*n*) and expression levels. Source data are provided as a Source Data file.

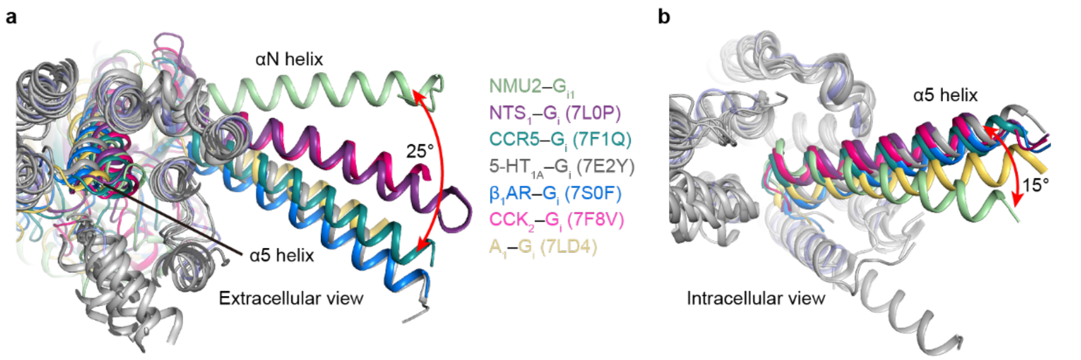

Supplementary Fig. 8 Comparison of the G protein coupling of NMU2–G_{i1} with other GPCR–G protein complexes. a, b Comparison of the G protein binding pose in NMU2–G_{i1}, NST_1–G_i (7L0P), CCR5–G_i (7F1Q), 5-HT_{1A}–G_i (7E2Y), $β_1$AR–G_i (7S0F) and CCK_2–G_i (7F8V) and A_1–G_i (7LD4) complexes. $Gα_i$ in NMU2–G_{i1}, NST_1–G_i, CCR5–G_i, 5-HT_{1A}–G_i, $β_1$AR–G_i and CCK_1–G_i and A_1–G_i are shown as cartoon and colored by pale green, violet purple, deep teal, gray, marine, hot pink and yellow orange respectively. NMU2 is shown as slate cartoon and other receptors are shown as gray cartoon. Conformational changes of αN helix (**a**) and α5 helix (**b**) are indicated by red arrows.

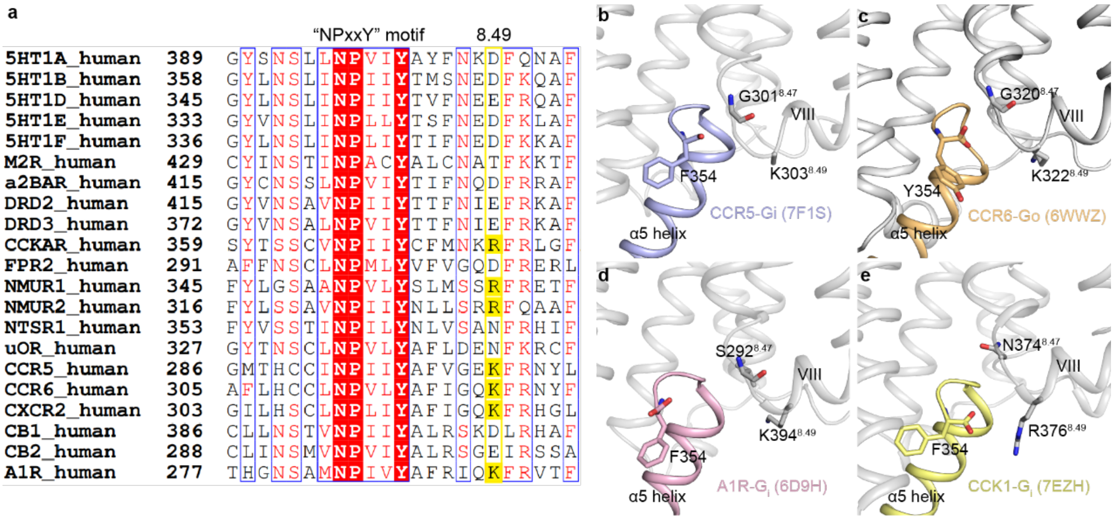

Supplementary Fig. 9 Interactions between the C terminus of $G_{i/o}$ and helix VIII of other GPCR-$G_{i/o}$ complexes. a Sequence alignment was prepared by GPCRDB and the graphic was generated by ESPript 3.0 server. Background colors represent the degree of similarity between different receptors: red, identical; red text, similar. The positively-charged residues in 8.49 are indicated by yellow square frames. **b-e** Interaction patterns between the C-terminal residues of $G_{i/o}$ and CCR5 (PDB ID: 7F1S), CCR6 (PDB ID: 6WWZ), A_1 (PDB ID: 6D9H) or CCK_1 (PDB ID: 7EZH). Receptors are shown as gray cartoon and G protein is indicated by light blue, wheat, light pink and light yellow cartoon respectively. Residues are shown as sticks.

Supplementary Table 1. Cryo-EM data collection, refinement and validation statistics of NmU-25–NMU2–G_{i1} complex structures.

	NMU-25–NMU2–G_{i1} (EMDB-33247) (PDB 7XK8)
**Data collection and processing**	
Magnification	81,000
Voltage	300
Electron exposure (e^{-}/ Å ^{2})	70
Defocus range (μm)	-0.8 ~ -1.5
Pixel size (Å)	1.045
Symmetry imposed	C1
Initial particle projections (no.)	3,088,911
Final particle projections (no.)	912,031
Map resolution (Å)	3.3
FSC threshold	0.143
Map resolution range (Å)	2.5 - 6.0
**Refinment**	
Initial model used (PDB code)	6OS9, 6LML
Model resolution (Å)	3.9
FSC threshold	0.5
Map sharpening B factor (Å^{2})	-136
Model composition	
Protein residues	923 (6,999 atoms)
Receptor residues	282 (2.318 atoms)
G protein residues	616 (4,478 atoms)
Ligand residues	25 (203 atoms)
B factors (Å^{2})	
Protein	49.1
Ligand	75.2
R.m.s. deviation	
Bond lengths (Å)	0.002
Bond angles (°)	0.465
Validation	
MolProbity score	1.60
Clashscore	7.55
Rotamer outlier (%)	0.00
Ramachandran plot	
Favored (%)	96.91
Allowed (%)	3.09
Disallowed (%)	0

Supplementary Table 2. NMU2 mediates the agonist-induced G protein activation tested by TRUPATH sensor.

NmU-25–induced G protein activation of NMU2									
Mutants[a]	EC_{50} (nM)	EC_{50} ratio[b]	pEC_{50} mean±SEM[c]	pEC_{50} *P* value	Span[c,d] (% of WT)	Span[c,d] *P* value	n[e]	Expression[f] (% of WT)	Expression[f] *P* value
WT	34	1	7.46±0.12	/	100±5	/	13	100	/
H43[1.30]E	360	11	6.44±0.16*	0.0399	135±8*	0.0291	4	84±7	0.2105
E102[2.61]A	nd	/	nd	/	/	/	4	57±5****	<0.0001
E105[2.64]A	2484	73	5.60±0.43****	<0.0001	65±13*	0.0291	4	46±3****	<0.0001
N109[ECL1]A	298	9	6.53±0.19	0.1444	138±11*	0.0374	3	98±3	0.9996
F126[3.32]A	625	18	6.20±0.83*	0.0177	27±10****	<0.0001	3	52±3****	<0.0001
E127[3.33]A	nd	/	nd	/	/	/	4	61±4****	<0.0001
H185[4.64]F	nd	/	nd	/	/	/	6	25±5****	<0.0001
W281[6.48]A	814	24	6.09±0.37**	0.0077	55±9**	0.0083	3	73±9**	0.0039
F284[6.51]A	nd	/	nd	/	/	/	5	32±3****	<0.0001
R288[6.55]A	nd	/	nd	/	/	/	4	31±7****	<0.0001
F291[6.58]A	737	22	6.13±0.19*	0.0106	126±11	0.3157	3	66±4***	0.0002
A302[7.28]H	162	5	6.79±0.28	0.5134	97±11	0.9997	3	78±5*	0.0343
F305[7.31]A	2603	77	5.58±0.26****	<0.0001	95±11	0.9995	4	97±6	0.9994
N306[7.32]Q	466	14	6.33±0.24*	0.0429	101±10	>0.9999	3	78±3*	0.0305
H309[7.35]A	nd	/	nd	/	/	/	4	25±6****	<0.0001
H309[7.35]F	nd	/	nd	/	/	/	3	25±6****	<0.0001
F316[7.42]A	1340	39	5.87±0.22***	0.0003	113±11	0.9108	4	80±4	0.0685

CPN 267-induced G protein activation of NMU2									
Mutants[a]	EC_{50} (nM)	EC_{50} ratio[b]	pEC_{50} mean±SEM[c]	pEC_{50} *P* value	Span[c,d] (% of WT)	Span[c,d] *P* value	n[e]	Expression[f] (% of WT)	Expression[f] *P* value
WT	144	1	6.84±0.46	/	0.022±0.005	/	9	100	/
H43[1.30]E	283	2	6.55±0.30	0.9267	0.049±0.007	0.1358	3	111±3**	0.0081
A302[7.28]H	/	/	/	/	0.015±0.009	0.8891	5	91±2*	0.0233
N306[7.32]Q	17	0.12	7.78±0.47	0.2626	0.041±0.009	0.1226	9	72±1****	<0.0001

CPN 116-induced G protein activation of NMU2									
Mutants[a]	EC_{50} (nM)	EC_{50} ratio[b]	pEC_{50} mean±SEM[c]	pEC_{50} *P* value	Span[c,d] (% of WT)	Span[c,d] *P* value	n[e]	Expression[f] (% of WT)	Expression[f] *P* value
WT	88	1	7.05±0.11	/	100±5	/	10	100	/
H43[1.30]E	3010	34	5.52±0.33****	<0.0001	68±10*	0.0441	3	94±2	0.8375
H43[1.30]W	328	4	6.48±0.18	0.1030	113±8	0.3894	4	82±2	0.0679
A302[7.28]H	890	10	6.05±0.42**	0.0048	57±12**	0.0043	3	78±5*	0.0284

N306$^{7.32}$Q	3030	34	5.52±0.17****	<0.0001	103±8	0.9887	4	78±3*	0.0260
N306$^{7.32}$W	nd	/	nd	/	/	/	3	96±10	0.9719

[a]Mutants represent that mutations were introduced separately into the WT receptor and transiently expressed in HEK 293F cells.

[b]The EC_{50} ratio was shown as $EC_{50(mutant)}/EC_{50(WT)}$, indicating the shift between the WT and mutant curves, reflecting the effect of the mutations on receptor signaling. Nd, not determined. /, not tested.

[c]Data are shown as mean ± S.E.M. from at least three independent experiments. One-way ANOVA was performed followed by Dunnett's post-test and compared with WT. The *P* value was defined as: *P<0.05; **P<0.01; ***P<0.001; ****P<0.0001.

[d] The span is defined as the window between the maximal agonists response (E_{max}) and minimal agonists response.

[e] Sample size; the number of independent experiments.

[f] Receptor expression level of HEK 293F cells was determined independently by flow cytometry with anti-FLAG antibody and shown as percent compared to the WT.

Supplementary Table 3. IP accumulation of WT and mutants NMU2 for NmU-25 and R-PSOP.

Mutants[a]	NmU-25		NmU-25/R-PSOP (10 μM)				Expression[f]	
	EC_{50} (nM) (pEC50±SEM)[b]	n[c]	EC_{50} (nM) (pEC50±SEM)	Ratio[d]	Ratio change (fold)[e]	n	(% WT)	*P* value
WT	17 (7.78±0.07)	13	215 (6.67±0.06)	13	1	13	100	/
E102$^{2.61}$A	1704 (5.77±0.09)	3	2563 (5.59±0.13)	2	6.5	5	55±4***	0.0002
E127$^{3.33}$A	2141 (5.67±0.18)	3	3804 (5.42±0.15)	2	6.5	3	61±4***	0.0006
F131$^{3.37}$L	11 (7.97±0.15)	3	31 (7.50±0.12)	3	4	3	111±10	0.5615
F177$^{4.56}$C	7 (8.15±0.13)	3	54 (7.27±0.13)	8	1.6	3	115±4	0.2510
F224$^{5.46}$A	46 (7.34±0.08)	3	165 (6.78±0.08)	4	3	4	150±8****	<0.0001
H285$^{6.52}$A	214 (6.67±0.13)	3	4238 (5.37±0.10)	20	0.65	4	54±4***	0.0001
R288$^{6.55}$A	nd	3	/	/	/	/	13±2****	<0.0001

[a]Mutants represent that mutations were introduced separately into the WT receptor and transiently expressed in HEK 293F cells.

[b]Data are shown as mean ± S.E.M. from three biological replicates. Nd, not determined. /, not tested.

[c]Sample size; the number of independent experiments.

[d]The EC_{50} ratio are shown as $EC_{50(NmU\text{-}25\ +\ R\text{-}PSOP)}/EC_{50(NmU\text{-}25)}$, indicating the shift between the NmU-25 and NmU-25 + 10 μM R-PSOP curves, reflecting the effect of antagonism on WT receptor or mutants.

[e]Ratio change indicates the EC_{50} ratio (WT)/EC_{50} ratio (mutant).

[f] Receptor expression level of HEK 293F cells was determined independently by flow cytometry with anti-FLAG antibody and shown as percent compared to the WT. Data are shown as mean ± S.E.M. from at least three independent experiments. One-way ANOVA was performed followed by Dunnett's post-test and compared with WT. The *P* value was defined as: **P*<0.05; ***P*<0.01; ****P*<0.001; *****P*<0.0001.

Supplementary references

1. Takayama, K. *et al.* Discovery of selective hexapeptide agonists to human neuromedin U receptors types 1 and 2. *J Med Chem* **57**, 6583-6593 (2014).
2. Takayama, K. *et al.* Discovery of a Human Neuromedin U Receptor 1-Selective Hexapeptide Agonist with Enhanced Serum Stability. *J Med Chem* **60**, 5228-5234 (2017).
