# Supplementary material for: Ligand recognition and activation of neuromedin U receptor 2
Source: Nat Commun. 2022 Dec 27;13:7955. doi: 10.1038/s41467-022-34814-4 (PMC9794833; doi:10.1038/s41467-022-34814-4)
Supplement: Supplementary file 3 — Reporting Summary [file 41467_2022_34814_MOESM3_ESM.pdf]

nature research

Corresponding author(s): Shuo Han, Beili Wu and Qiang Zhao

Last updated by author(s): Oct 30, 2022

Reporting Summary

Nature Research wishes to improve the reproducibility of the work that we publish. This form provides structure for consistency and transparency in reporting. For further information on Nature Research policies, see our Editorial Policies and the Editorial Policy Checklist.

Statistics

For all statistical analyses, confirm that the following items are present in the figure legend, table legend, main text, or Methods section.

n/a	Confirmed	
☐	☒	The exact sample size (*n*) for each experimental group/condition, given as a discrete number and unit of measurement
☐	☒	A statement on whether measurements were taken from distinct samples or whether the same sample was measured repeatedly
☐	☒	The statistical test(s) used AND whether they are one- or two-sided<br>*Only common tests should be described solely by name; describe more complex techniques in the Methods section.*
☒	☐	A description of all covariates tested
☒	☐	A description of any assumptions or corrections, such as tests of normality and adjustment for multiple comparisons
☐	☒	A full description of the statistical parameters including central tendency (e.g. means) or other basic estimates (e.g. regression coefficient) AND variation (e.g. standard deviation) or associated estimates of uncertainty (e.g. confidence intervals)
☐	☒	For null hypothesis testing, the test statistic (e.g. F, t, r) with confidence intervals, effect sizes, degrees of freedom and P value noted<br>*Give P values as exact values whenever suitable.*
☒	☐	For Bayesian analysis, information on the choice of priors and Markov chain Monte Carlo settings
☒	☐	For hierarchical and complex designs, identification of the appropriate level for tests and full reporting of outcomes
☒	☐	Estimates of effect sizes (e.g. Cohen's *d*, Pearson's *r*), indicating how they were calculated

Our web collection on statistics for biologists contains articles on many of the points above.

Software and code

Policy information about availability of computer code

Data collection	Automated data collections on the Titan Krios was performed using serialEM 3.7.
Data analysis	The following softwares were used in cryo-EM data processing, model building and structure validation: MotionCor2, Gctf v1.06, RELION-3, UCSF Chimera 1.3.1, COOT 0.8.9, and Phenix 1.15.2, MolProbity 4.2.<br>The functional data were analyzed by GraphPad Prism 8.0.<br>The figures were prepared using pymol 2 and UCSF Chimera 1.3.1.

For manuscripts utilizing custom algorithms or software that are central to the research but not yet described in published literature, software must be made available to editors and reviewers. We strongly encourage code deposition in a community repository (e.g. GitHub). See the Nature Research guidelines for submitting code & software for further information.

Data

Policy information about availability of data

All manuscripts must include a data availability statement. This statement should provide the following information, where applicable:

- Accession codes, unique identifiers, or web links for publicly available datasets
- A list of figures that have associated raw data
- A description of any restrictions on data availability

Atomic coordinate and the cryo-EM density map of NmU-25–NMU2–Gi1 complex has been deposited in the RCSB Protein Data Bank (PDB) under accession code 7XK8, and Electron Microscopy Data Bank (EMDB) under accession code EMD-33247. All relevant data are available from the corresponding authors upon resonable request. Source data are provided with this paper. The database used in this study includes PDB 6OS9, 7F9Y, 4BUO, 6LFO, 7EZH, 6D9H, 6N4B, 7BZ2, 7E32, 7CKZ, 7BU6, 7L0P, 7E2Y, 7S0F, 7F8V, 7LD4, 7F1S, 6WWZ, 6D9H, 7EZH,

Field-specific reporting

Please select the one below that is the best fit for your research. If you are not sure, read the appropriate sections before making your selection.

☒ Life sciences ☐ Behavioural & social sciences ☐ Ecological, evolutionary & environmental sciences

For a reference copy of the document with all sections, see nature.com/documents/nr-reporting-summary-flat.pdf

Life sciences study design

All studies must disclose on these points even when the disclosure is negative.

Sample size	All functional data were obtained from at least three independent experiments. Sample size for the cryo-EM studies was determined by availability of microscope time and to ensure unambiguous modeling of the structures.
Data exclusions	No data were excluded from the analyses.
Replication	All functional assays were performed in triplicate. All attempts at replication were successful.
Randomization	Randomization is not relevant to this study, as protein samples are not required to be allocated into experimental groups in protein structural studies, and no animals or human research participants are involved in this study. TRUPAT and IP accumulation assay did not allocate experimental groups as well.
Blinding	Blinding is not relevant to this study, as no subjective allocation was involved in any of the structural and functional experiments.

Reporting for specific materials, systems and methods

We require information from authors about some types of materials, experimental systems and methods used in many studies. Here, indicate whether each material, system or method listed is relevant to your study. If you are not sure if a list item applies to your research, read the appropriate section before selecting a response.

Materials & experimental systems

n/a	Involved in the study	
☐	☒	Antibodies
☐	☒	Eukaryotic cell lines
☒	☐	Palaeontology and archaeology
☒	☐	Animals and other organisms
☒	☐	Human research participants
☒	☐	Clinical data
☒	☐	Dual use research of concern

Methods

n/a	Involved in the study	
☒	☐	ChIP-seq
☐	☒	Flow cytometry
☒	☐	MRI-based neuroimaging

Antibodies

Antibodies used	Cryptate-labelled anti-IP1 monoclonal antibody (clone name: IP1 Tb Cryptate Antibody): CisBio Bioassays, Cat#62IPAPEC, 1:20 diluted in lysis and detection buffer;<br>Anti-FLAG M2-FITC antibody (clone name: M2, monoclonal): Sigma, Cat#F4049, 1:100 diluted in TBS+4% BSA.
Validation	All antibodies were commercially obtained and validation reports are available on the supplier website:<br>Cryptate-labelled anti-IP1 monoclonal antibody: https://www.cisbio.cn/ip-one-gq-kit-40451#section-products-tabs-product;<br>Anti-FLAG M2-FITC antibody: https://www.sigmaaldrich.com/technical-documents/articles/biofiles/antibodies-to-peptides.html.

Eukaryotic cell lines

Policy information about cell lines

Cell line source(s)	The Hi5 and HEK293F cell lines were originally obtained from Invitrogen.
Authentication	None of the cell lines have been authenticated.
Mycoplasma contamination	The cell lines were negative for mycoplasma contamination.
Commonly misidentified lines (See ICLAC register)	No commonly misidentified cell lines were used.

Flow Cytometry

Plots

Confirm that:

- [ ] The axis labels state the marker and fluorochrome used (e.g. CD4-FITC).
- [ ] The axis scales are clearly visible. Include numbers along axes only for bottom left plot of group (a 'group' is an analysis of identical markers).
- [ ] All plots are contour plots with outliers or pseudocolor plots.
- [ ] A numerical value for number of cells or percentage (with statistics) is provided.

Methodology

Sample preparation	Expression level of the receptor was measured by incubating 10µl cells with 15 µl TBS buffer supplemented with 4% BSA, 20% (v/v) viability staining solution7-AAD (Invitrogen, Cat#00-6993-50) and ANTI-FLAG M2-FITC antibody (Sigma, F4049; 1:100 diluted by TBS) at 4 °C for 20 min. After incubation, 175µl TBS buffer was added and the fluorescence signal on the cell surface was detected by a FCM (flow cytometry) reader (Millipore).
Instrument	Guava easyCyte HT, Millipore
Software	GuavaSoft 2.2.2, Guava ExpressPlus panel.
Cell population abundance	For each measurement, 2,000 cell events were collected and the fluorescence intensity of cell population with protein expression was calculated.
Gating strategy	Gating was determined by the Green-red fluorescence intensity to differentiate positive cells.

- [ ] Tick this box to confirm that a figure exemplifying the gating strategy is provided in the Supplementary Information.
